# Regulation of sleep by KIN-29 is not developmental

**DOI:** 10.17912/micropub.biology.000247

**Published:** 2020-05-07

**Authors:** Jeremy J. Grubbs, Alexander M. van der Linden, David M. Raizen

**Affiliations:** 1 Department of Neurology, Perelman School of Medicine, University of Pennsylvania, Philadelphia, Pennsylvania 19104; 2 Department of Biology, University of Nevada, Reno, Reno, Nevada 89557

**Figure 1:**
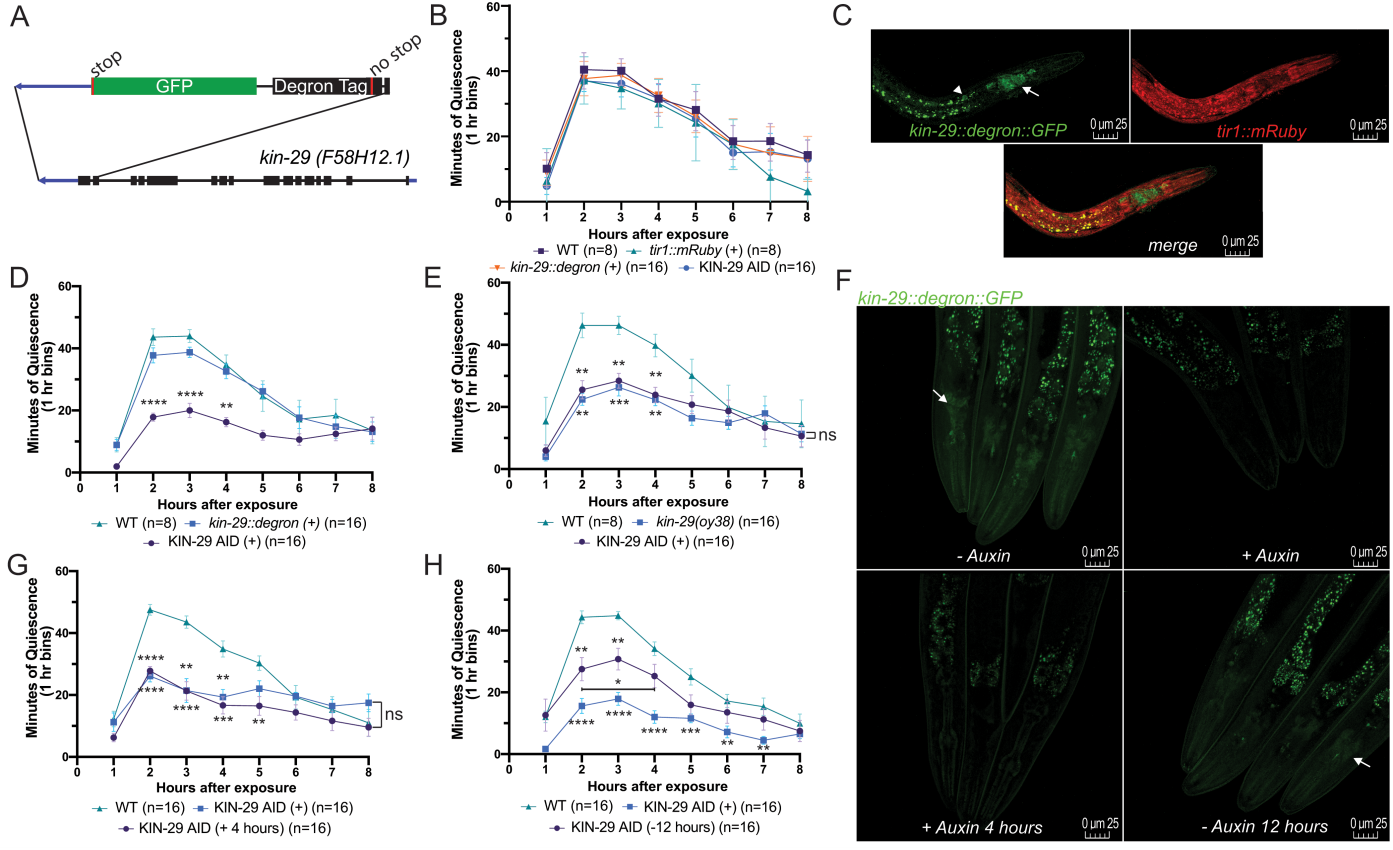
**Regulation of sleep by KIN-29 is not developmental. A)** Schematic of the CRISPR/Cas9 strain construction with the DEGRON and GFP-tags inserted at the end of the *kin-29* open reading frame.For panels B, D, E, G and H, the average movement quiescence in minutes ± SEM are displayed following exposure to 1500 J/m^2^ UVC irradiation. (+) indicates that 2 mM auxin was present during development and analysis. **B)** UVC–SIS is similar to wild type (WT) in auxin–exposed *tir1*::*mRuby* and *kin-29::degron::GFP* animals, and in *tir1::mRuby*; *kin-29::degron::GFP* (KIN-29 AID)transgenic animals in the absence of auxin. **C)** An image of a KIN-29 AID L4 stage animal. GFP is expressed in head neurons (white arrow) and in the intestine (white arrow head) consistent with previous reports (Lanjuin and Sengupta 2002). *tir1::mRuby* is expressed ubiquitously, as reported (Zhang *et al.* 2015). **D)** UVC–SIS is impaired in KIN-29 AID animalsexposed to auxin from the time of hatching. **E)** The UVC–SIS impairment produced by continuous auxin–induced degradation of KIN-29 is similar to that of *kin-29(oy38)* null mutants. **F)** Images of adult KIN-29 AID animals never exposed to auxin (*top left*), exposed to auxin since hatching (*top right*), exposed to auxin for 4 hours as adults (*bottom left*), and removed from auxin for 12 hours starting at the L4 stage after exposure from hatching (*bottom right*). White arrows point to head neurons (green), which are visible only in the absence of auxin. Images were captured with identical camera and microscope settings. **G)** KIN-29 AID animals show a similar defect in UVC–SIS whether exposed to auxin at hatch or from the adult stage for four hours. **H)** UVC–SIS in KIN-29 AID animals on auxin from hatching through adulthood (+) or until the L4 stage, and examined 12 hours later (-12 hours). Reduction of auxin-induced degradation of KIN-29 during adulthood partially restores SIS behavior. ns = not significant, *P<0.05, **P<0.01, ***P<0.001, ****P< 0.0001, two-way ANOVA or mixed-model analysis with Tukey correction for multiple comparisons. Comparisons are made against wild-type (WT) unless otherwise indicated.

## Description

Sleep is intertwined with metabolic function in vertebrates (Tsuneki *et al.* 2016; Herrera *et al.* 2017; Aalling *et al.* 2018; Wilms *et al.* 2018) and invertebrates (Kempf *et al.* 2019; Ki and Lim 2019; Yurgel *et al.* 2019; Grubbs *et al.* 2020), but the molecular underpinnings of this connection are not well understood. We recently reported that the salt inducible kinase (SIK) homolog KIN-29 is required in a subset of sensory neurons for the metabolic regulation of sleep in *Caenorhabditis elegans* (Grubbs *et al.* 2020). However, since our genetic manipulations made use of mutations that were present throughout the life of the animal, it is possible that the sleep defect of *kin-29* mutants reflects a requirement for KIN-29 activity during development rather than during sleep. Indeed, because *kin-29* is expressed throughout development as well as adulthood (Maduzia *et al.* 2005), and *kin-29* mutants show altered expression of targets influential in larval development (Van Der Linden *et al.* 2008), a role for *kin-29* during development remained plausible. Distinguishing a developmental early role from a role during the time of sleep is important for constraining models for how KIN-29 regulates sleep.

To determine the KIN-29 time window of action, we depleted the KIN-29 protein in a temporally-controlled fashion. We used CRISPR/Cas9 genome editing to introduce a DEGRON fused to GFP just before the *kin-29* stop codon (**Fig. 1A**). After confirming successful editing by Sanger sequencing, we crossed the *kin-29::degron::GFP* into the previously generated *Peft-3::TIR1::mRuby* strain (Zhang *et al.* 2015) to allow for rapid and reversible degradation of KIN-29 when exposed to the phytohormone auxin (Ruegger *et al.* 1998; Gray *et al.* 1999). We examined stress-induced sleep (SIS) following exposure to UVC-irradiation (Debardeleben *et al.* 2017) in the first day of adulthood. We first verified that auxin exposure did not disrupt SIS in the parental strains, and that the progeny containing both transgenes, from here on referred to as KIN-29 Auxin-Inducible Degron (KIN-29 AID) animals, show normal SIS in the absence of auxin (**Fig. 1B**). We also confirmed the expression of both transgenes in KIN-29 AID animals (**Fig. 1C**). We then measured SIS in KIN-29 AIDtransgenic worms cultivated on agar containing auxin (2 mM) from the time of hatching. KIN-29 AID transgenic animals cultivated on auxin throughout development mimicked the *kin-29(oy38)* null mutant SIS phenotype (**Fig. 1D-E)**.Such animals also showedundetectable expression of endogenous GFP-tagged KIN-29 (**Fig. 1F**).

To determine if KIN-29’s regulation of sleep has a developmental component, we used two paradigms. First, we cultivated young adult animals for four hours on agar containing auxin (2 mM). We then administered a UVC treatment (1500 J/m^2^) and recorded movement quiescence of animals in the continued presence of auxin. Four hours in the presence of auxin at the young adult stage was sufficient to reduce GFP-tagged KIN-29 expression (**Fig. 1F**), and was able to reduce SIS to a similar extent as that seen in animals cultivated on auxin their whole life (**Fig. 1F-G**). Second, to restore KIN-29 function to adult animals, we cultivated KIN-29 AID transgenic animals in the presence of auxin since hatching, but then removed them from auxin-containing plates at the L4 stage. We waited twelve hours before assessing SIS using UVC-irradiation treatment. These animals showed significantly increased movement quiescence in comparison to animals that were cultured their whole life on auxin, although these quiescence levels were lower than the quiescence phenotype of *wt* animals (**Fig. 1H**). Consistent with these intermediate behavioral results, expression of GFP-tagged KIN-29 in these animals was intermediate between that of animals never exposed to auxin, and that of animals exposed to auxin throughout life (**Fig. 1F**). Our results are consistent with previous observations that even after 24 hours off of auxin, expression of a DEGRON-tagged protein may not fully normalize (Zhang *et al.* 2015).

In summary, our results demonstrate that KIN-29’s function in SIS is not developmental. Rather, these findings suggest that *kin-29* functions at the time of sleep. These results fit with our proposed model of *kin-29* acting as a real-time energy sensor in sensory neurons to promote sleep (Grubbs *et al.* 2020). Based on this analysis, we propose that the sleep-regulatory effects of SIK3 in mammals (Funato *et al.* 2016) are also unlikely to be developmental.

## Methods

**Worm maintenance and strains**

Worms were cultivated on the agar surface of 5.5 cm diameter Petri dishes filled with 11.5 mL of NGM agar. They were fed the *Escherichia coli* strain DA837 (Davis *et al.* 1995) and grown at 20°C. All experiments were performed on hermaphrodites. The wild-type strain used was N2, variety Bristol (Brenner 1974). Auxin experiments were carried out as described in (Zhang *et al.* 2015) using 2 mM auxin (Alfa Aesar #A10556). Strains available from the CGC are N2 and CA1200. Strains available from our lab are PHX1777 *kin-29*(*syb1777*[*kin-29::degron::gfp*]) and NQ1295 (ieSi57[*Peft-3*::*tir1*::*mRuby*]; *kin-29(syb1777*[*kin-29::degron::gfp*]).

**CRISPR/Cas9 genome editing and crosses**

CRISPR/Cas9-mediated genome editing was performed by SunyBiotech (China). The oligonucleotide sequence used for PCR and Sequencing were (Sense) 5’CATTGTGTGCACATTACCGT and (Antisense) 5’AATCAGCTAGCACAGGCTCT. To generate the strain NQ1295, PHX1777 males were crossed with CA1200 hermaphrodites, with the red and green fluorescent phenotypes followed using a Leica SP8 confocal microscope. We found a regular epifluorescence compound microscope not sufficiently sensitive to detect the fluorescence in these strains, which was very dim.

**Ultraviolet light exposure and movement quiescence recordings**

Movement quiescence was quantified using machine vision with images recorded using the 48-well WorMotel (Churgin *et al.* 2019). Briefly, animals were selected as L4 larvae 12-16 hours prior to behavioral recording. All 48 wells were filled with NGM agar with or without 2 mM auxin, which was diluted from a 400 mM stock as described in (Zhang *et al.* 2015), and a thin layer of bacteria was spread over the agar surface using a platinum wire. Animals were loaded individually as adults into WorMotel wells and then treated using a Spectrolinker XL-1000 crosslinker with 1500 J/m^2^ of UVC (254 nm) radiation. The WorMotel was placed under a DMK 23GP031 camera (The Imaging Source) for recording. Images were captured every 10 seconds for 8 hours. Images collected of the animals on the WorMotel were analyzed with custom MatLab scripts (Churgin *et al.* 2019). The quantification of movement was determined via pixel subtraction and an animal was considered quiescent if there was no pixel change between images.

**Confocal imaging**

Animals were placed in a 1ml droplet of M9 buffer and immobilized on a 2% agarose pad containing 25mM levamisole. Images were taken using a Leica SP8 confocal microscope as Z-stacks with 0.76 microns between images in a stack and a total of 36 images per condition taken. The powers of the 488 nm and 552 nm lasers were set to 2.5% and 2% respectively. Z-projection was performed in FIJI (Version 2.0.0).
